# Blends of Salvinia molesta oil microemulsion with diesel in an unmodified diesel engine for the simultaneous reduction of nitrogen oxide and smoke

**DOI:** 10.1016/j.heliyon.2024.e30681

**Published:** 2024-05-03

**Authors:** Marutholi Mubarak, Andavan Shaija, Tharamel Vasu Suchithra

**Affiliations:** a Department of Mechanical Engineering, MEA Engineering College Perinthalmanna, Malappuram, 679325, India; b Department of Mechanical Engineering, National Institute of Technology, Calicut, 673601, India; c School of Biotechnology, National Institute of Technology, Calicut, 673601, India

**Keywords:** Brake thermal efficiency, Microemulsion, Peak pressure, Smoke

## Abstract

In this study, microemulsion synthesized from chemically extracted *Salvinia molesta* oil with diesel was evaluated as fuel in stationary unmodified diesel engine. The microemulsions from *S. molesta* oil was prepared using the best combinations of 67% *S. molesta* oil, 15% ethanol, 13% water and 5% surfactant (span 80) and its properties were compared with that of diesel. The engine test conducted with M10, M20 and M30 blends and reported a brake thermal efficiency of 29.76% and brake specific fuel consumption of 0.3239 kg/kWh with M20. The emissions like NO and smoke reduced by 18.07% and 7.37%, respectively, with marginal increase in CO, CO_2_ and unburned hydrocarbon by 3.8%, 3.4% and 16.66% respectively, with M20 compared to diesel at maximum engine load of 3.73 kW. At lower engine loads with M10, M20 and M30 slightly lower CO_2_ emission than diesel. A drop in peak pressure and heat release rate was found to be 1.73% and 8.40%, correspondingly with M20, as that of diesel. Even though a slight reduction in brake thermal efficiency observed with M20 as compared to M10 and diesel by considering the lowest emissions of NO and smoke, it is feasible to use as promising fuel for unmodified diesel engines.

## 1 Introduction

The increasing prices of the fast-depleting fossil fuels and the effect of its usage on the atmosphere have led to the search for unconventional fuels for diesel engines [ [1,2]]. The incessantly used in different applications such as transportation, agricultural and power generation [3,4]. The biofuels have potential to provide a reliable and cost-effective alternative to the increasing energy demands of diesel engines [5,6]. Biofuels such as biodiesel, microemulsions are the promising unconventional fuels to use in diesel engines due to its reduced tail pipe emissions, unburned hydrocarbon, particulate matter, polyaromatics, renewability and biodegradability [7]. Biofuels are produced from different feedstocks such as Jatropha, Pongamia, rapeseed, waste cooking oil [[8], [9], [10]] but this are limited due to less availability of land for cultivation, causing food versus energy competition [11]. The usage of third generation biofuels such as microalgae and aquatic weeds like *Salvinia molesta* avoids the problem of food and land versus energy competition and doesn't require any artificial cultivation [ [[12], [13], [14]]]. Kariba weed, also known as *S. molesta*, is a floating plant found in most freshwater bodies, including lakes, ponds, and dams. *S. molesta* primarily causes obstructions in hydroelectric dams, limits irrigation, creates flooding and erosion, lessens the amount of suitable habitat for native fish, including eels and whitebait, and renders the water unfit for human consumption [7,11]. Biofuels produced from microalgae and aquatic weeds like *Salvinia molesta* are promising due to high areal productivity, renewability, and high lipid content [15,16]. The steps involved in the biofuel production from *S. molesta* are its collection, drying and powdering, extraction of lipid, and it's processing [17]. Out of these steps, extraction of lipid is an important step as it is highly energy intensive. The extraction of lipid using solvent extraction method is most promising one due to its simplicity and higher efficiency [18].

The common techniques for using the extracted lipid or oil as a fuel in diesel engines are blending with diesel, transesterification, pyrolysis, and micro emulsification [19]. The blending of raw oil with diesel is not desirable due to its higher viscosity, which leads to improper fuel atomization causing higher exhaust emissions [20]. The method of transesterification process involves complex time-consuming chemical reactions, which makes it an expensive process. Pyrolysis requires expensive equipment for carrying out the reactions [21,22]. A simple and successful way of utilizing raw oil in engine is by using its micro emulsions blends [23]. Micro emulsions are micro-heterogeneous, thermodynamically stable and spontaneously formed mixture of oil, water and alcohol with surfactants [24]. The hydrophile-lipophile balance (HLB) for screening appropriate non-ionic surfactants, low HLB surfactants are more effective for stabilizing water-in oil emulsion and for stabilizing oil in water, and emulsion high HLB surfactants are used. The most commonly used surfactant is span 80 with 4.3 HLB having thermal sensitive agents with more intensive hydrophobic behavior at higher temperatures [25]. By adding water, combustion temperature reduces effectively restraining NOx emissions and micro-explosion action of water improves the spraying effects [[26], [27], [28]]. The microemulsion prepared using methanol, ethanol, butanol can reduce the viscosity of the microalgal oil as required for the diesel fuel. Microemulsification has been considered as one of the reliable approaches to solve the problem of the high viscosity of vegetable oils [29]. Also, the usage of microemulsion doesnot require any modifications in fuel supply system and on the engines [30].

The combustion of microemulsion occurs with the phenomenon of micro explosion which is due to the simultaneous evaporization of the water droplets in the fuel droplet as the fuel is baring to growing in-cylinder temperature while injection which outcomes in significant drop in emission of smoke in diesel engines [31,32].

A few works have been reported in literature using microemulsion produced from vegetable oils for analysing the performance, emission and combustion characteristics of diesel engines. Kumar and Jaikumar [31] used microemulsion prepared from waste cooking oil as diesel engine fuel and reported significant reduction in NOx, as compared to diesel. Akechai Sankumgon et al. [33] produced microemulsions form crude jatropha oil, diesel and ethanol with the presence of surfactant. They reported that fuel properties and are comparable with diesel standard. The engine test results indicated comparable engine performance with lower smoke emission. Thu Nguyen et al. [34] formulated microemulsion from canola oil with ethanol and butanol and oleyl amine as surfactant. They reported that the fuel properties are comparable with ASTM requirements with higher fuel conusmption in engines with significant reduction in particulate emission. Najjar et al. [35] studied the emission characteristics of microemulsion based on sunflower oil, waster, butanol and Span 80 as surfactant. They reported decreased specific fuel consumption with reduced CO and NOx emissions as compared to neat diesel.

The purpose of this research work is to assess the capability of using microemulsion produced from *Salvinia molesta* oil as fuel to study the engine characteristics such as performance, emission, and combustion evaluation for the simultaneous reduction of emissions of nitrogen oxide and smoke of single cylinder stationary diesel engine. Only very limited studies are available with microemulsion as fuel in diesel engine and this is the first study reported with *Salvnia molesta* microemulsion. This work having three components; (i) Production of *Salvinia molesta* microemulsion from the lipid extracted (ii) Fuel properties of *Salvinia molesta* microemulsion blends prepared with diesel (iii) Experimental analysis to analyze the engine features using blends of *S. molesta* microemulsion with diesel.

## 2 Techniques and protocols for experiments

### 2.1 Fuel and experimental set up

Separation of lipid from *Salvinia molesta* for producing microemulsion.

*S. molesta* was collected from freshwater bodies closer to Mavoor, Calicut, India and washed thoroughly and sundried for two weeks. Lipid was then extracted, from the dried and powdered *S. molesta* using Soxhlet apparatus with a solvent to biomass ratio, temperature and time as 20:1 (vol./dry wt.), 85^o^C and 137 min, respectively based on author's previous study [36]. Chloroform: methanol (1:2 v/v) used as the solvent for extracting lipid in Soxhlet apparatus. The solvent lipid mixture obtained from Soxhlet apparatus was used in rotary vacuum evaporator for removing the chloroform and methanol. Then *S. molesta* microemulsion was prepared from S. molesta oil by mixing it with ethanol as co-surfactant, water and surfactant as span 80. Different combinations of microemulsion mixtures were prepared by varying *S. molesta* oil (65%–70%), ethanol (10%–15%), water (10%–17%) and surfactant (5%–10%) in % by volume to find the best stable combination. Each component was mixed thoroughly using magnetic stirrer to form a homogeneous phase. The various formulations of emulsion formed were kept for stability analysis at room temperature for 48 h. From the stability analysis, the best stable combination of microemulsion was found as 67% oil, 15% ethanol, 13% water and 5% surfactant as shown in Table 1. 300 mL of *S. molesta* oil microemulsion (M100) was then prepared by adding 39 mL water with 45 mL ethanol and 201 mL *S. molesta* oil with 15 mL of surfactant.

**Table 1:** Composition of best *S.molesta* microemulsion.

Component name	Percentage
*S.molesta* oil	67%
Ethanol	15%
Water	13%
Surfactant (span 80)	5%

### 2.2 Fuel properties of *S.molesta* microemulsion

Using the instruments and ASTM protocols mentioned in the authors' prior study, *S. molesta* microemulsion fuel characteristics and its combination with diesel were examined [7]. In three different blends of diesel, 10% volume *S. molesta* microemulsion plus 90% volume diesel (M10), 20% volume *S. molesta* microemulsion plus 80% volume of diesel (M20), and 30% volume *S. molesta* microemulsion plus 70% volume diesel (M30), the best stable microemulsion was used. As shown in Table 2, M100's specific gravity of 0.890 satisfies the ASTM 6751-02 standard's threshold, and its blends are found to have closer values of diesel specific gravity. M100 has a kinematic viscosity of 5.902 mm^2^/s, substantially within the range (1.9–6 mm^2^/s) of ASTM 6751-02 standard and for its blends are found closer to diesel and above the ASTM D975. The calorific value of M100 is 34.950 MJ/kg, which is comparable with that of diesel. The reduced calorific value of M100 is due to the presence of ethanol and water in the fuel. Also, M10, M20 and M30 showed calorific values in MJ/kg were 39.045, 38.332 and 36.932, respectively which are comparable with diesel. The comparatively reduced calorific value for M30 is due to the reduced percentage by volume (70%) of diesel present in it. The copper strip corrosion of M100 is found to be 1b which is within the threshold of ASTM standard (<3a) and it satisfies for its blends. The flash and fire points of M10, M20 and M30 were found to be closer with diesel. The cloud and pour point of M100 and its blends are to be within the limit specified by the ASTM standard. The cold flow properties of M10, M20 and M30 were found improved as compared to neat diesel due to the presence of ethanol in the microemulsion composition. The presence of ethanol in the fuel improves the cold flow property [37]. Thus, blends of M100 can be used as fuel in diesel engine.

**Table 2:** Evaluation of the *S. molesta* microemulsion's and its blends' physical and chemical characteristics.

Properties	*S.molesta* oil [7]	M100	M10	M20	M30	Diesel	ASTM D975	ASTM 6751-02
Specific gravity	**0.910**	**0.890**	0.831	0.839	0.849	0.821	–	0.870-0.890
Kinematic viscosity @ 40^o^C, mm^2^/s	**15.3**	**5.902**	3.812	4.12	4.63	2.716	1.9- 4.1	1.9-6
Calorific value, MJ/kg	32.500	34.95	39.04	38.33	36.93	42.200		–
Copper strip corrosion, 50^o^C for 3 h	1b	1b	1b	1b	1b	1a	<3a (dark tarnish)	<3a (dark tarnish)
Flash Point, ^o^C	–	124	93	96	101	79	52	>130
Fire Point, ^o^C	–	131	98	102	107	86		>130
Cloud point, ^o^C	–	−6	−8	−9	−10	−5	−15 to +5	−15 to +5
Pour point, ^o^C	–	−9	−12	−13	−15	−17	−35 to −15	−35 to −15
Cold filter plugging point, ^o^C	–	−10	−13	−15	−16	−8		–

The performance, emission and combustion features of the selected diesel engine was analyzed using different blends of *S. molesta* oil microemulsion (M100) as M10, M20 and M30, and was compared with diesel as fuel.

### 2.3 Engine set up

As shown in Table 3 naturally articulated stationery, constant speed variable load water-cooled, and four stroke single cylinder diesel engine connected with eddy current dynamometer was used for this study. The engine was loaded maximum at 3.73 kW for this study with a step of 0.98 kW, 1.98 kW, 2.98 kW and 3.73 kW. An AVL DiGas 444 five gas analyzer was employed for measuring five exhaust gases such as unburned hydrocarbon (HC), Carbon monoxide (CO), nitric oxide (NO), oxygen (O_2_), carbon dioxide (CO_2_) along with a AVL 437C smoke meter for measuring the smoke opacity of the exhaust gas. The engine in-cylinder pressure was measured using a pressure transducer (Kistler 6055 sensor) and instantaneous crank angles are recorded with an encoder. An injection timing of 23^o^bTDC was set for entire experimental work.

**Table 3:** Details of the engine testing apparatus.

Engine make, model	Kirloskar, AV_1_
Number of cylinders	1
Cylinder bore/stroke	80 mm/110 mm
Compression ratio	17.5:1
Rated speed	1500 rpm
Maximum power	3.73 kW
Injection timing	23^o^bTDC
Injection pressure	200 bar
Number of holes in nozzle	3
Torque	23 Nm
Dynamometer type	Eddy current
Dynamometer cooling	Air cooled
Capacity of dynamometer	5.5 kW

### 2.4 Uncertainty analysis

The accuracy of the experiments is able to identify with the help of uncertainty analysis by considering instrument state, calibration, environment and planning of test. The error in the measurement of parameter is the main reason of error in experiments. The total uncertainties in percentage in experiments is calculated with the principle of propagation of errors as shown in Eq. (1) [7,38,39].

(1) Total uncertainty in percentage = (Uncertainty of (BP^2^ + BTE^2^ + BSFC^2^ + NO^2^ + CO^2^ + CO_2_^2^ + UBHC^2^ + O_2_^2^+ EGT^2^ + smoke^2^ +Time^2^+ TDC^2^ +pressure^2^))^0.5^

= 1.5556%

The engine, exhaust gas analyzer, smoke meter, and other devices used in this experimental work have their uncertainties listed in Table 4. Using M10, M20, and M30 and pure diesel as fuel, the engine test was run at constant speed, variable load, and injection pressure to examine the performance, emissions, and combustion characteristics.

**Table 4:** Engine and other instrument uncertainties [7].

Species	Uncertainty (%)
CO	±0.3
CO_2_	±0.2
HC	±0.2
O_2_	±0.3
NO	±0.2
Smoke opacity	±0.1
EGT	±0.1
Digital stop watch	±0.2
Pressure	±0.1
TDC marker	±0.2
Brake power	±0.1
BSFC	±1
BTE	±1

## 3 Results and discussions

The engine characteristics curves were plotted based on constant speed variable load test along with emission and combustion study of single cylinder diesel engine using M10, M20 and M30 and pure diesel as fuel.

### 3.1 Performance analysis

#### 3.1.1 Brake thermal efficiency (BTE)

**Fig. 1:**
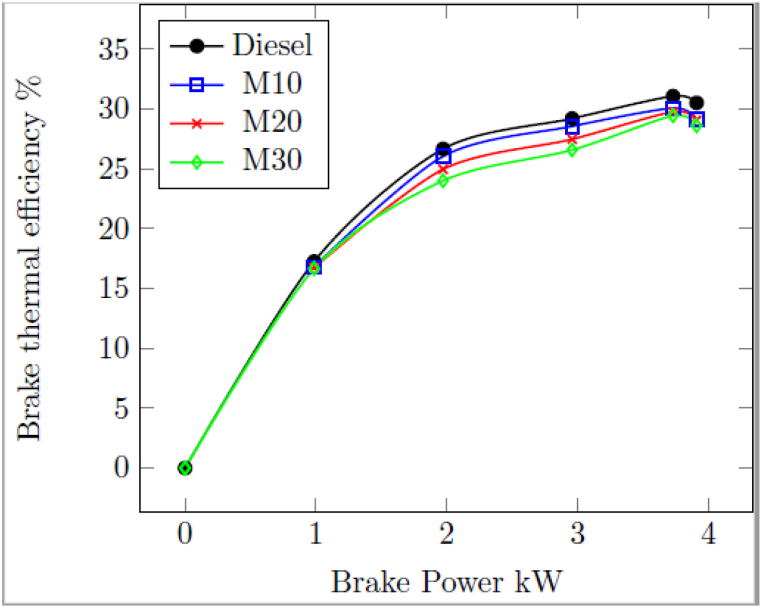
Variation of BTE with BP.

Fig. 1 indicates the variation of BTE of tested engine fuelled with M10, M20 and M30 and diesel for different loads. All fuels showed lesser BTE than neat diesel. Due to the lower heating values of M10, M20, and M30, the diesel had a BTE of 30.71% at the highest load of 3.73 kW, compared to 30.06% with M10, 29.76% with M20, and 29.43% with M30. Higher specific gravity and lower heating value are the main causes of blended fuels' lower brake thermal efficiency [23]. The higher specific gravity and viscosity tends to improve the fuel inline pressure [40] due to advanced injection of fuel in the combustion chamber with bulk modulus effect. The higher specific gravity of the fuel leading to poor atomization and higher energy for pumping is essential [41].

#### 3.1.2 Brake specific fuel consumption (BSFC)

**Fig. 2:**
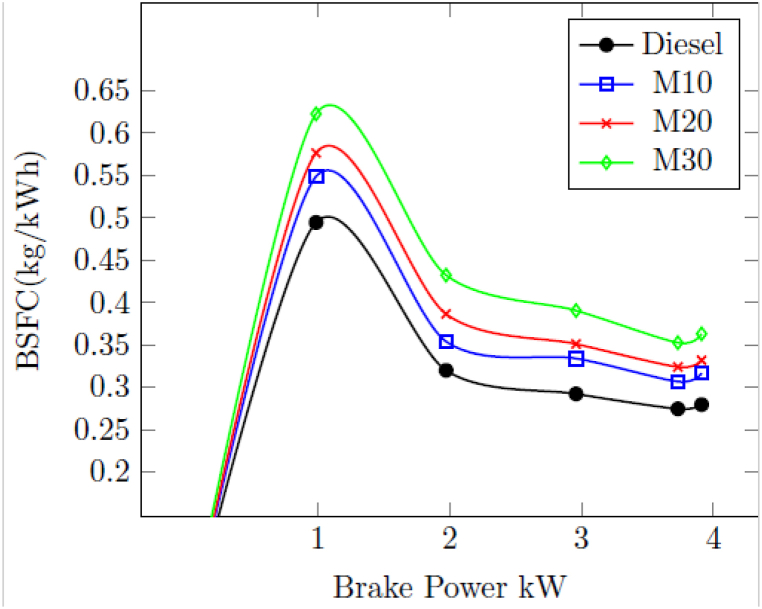
Change in BSFC with BP.

Fig. 2 indicates the change in BSFC of tested engine with M10, M20, M30 and pure diesel as fuel with different loads. Compared to pure diesel, all blended fuels result in higher BSFC. When 3.73 kW was the maximum load, the diesel-powered BSFC displayed 0.2745 kg/kWh; this value increased to 0.3066 kg/kWh with M10, 0.3239 kg/kWh with M20, and 0.3524 kg/kWh with M30. It was found that the blended fuels' higher specific gravity and lower heating value than diesel account for their higher BSFC. Since BSFC is calculated on a weight basis, fuel consumption is higher when fuel is used on a volume basis. The diesel engine BSFC value depends upon the relative ratio of parameters such as viscosity, density, volume based fuel injection system and lower heating value. As the heating value of M10, M20 and M30 is lower, a larger amount is needed to get injected a various loads to compensate chemical energy of the fuel and to achieve higher loads.

### 3.2 Analysis of emissions

#### 3.2.1 Carbon monoxide (CO)

**Fig. 3:**
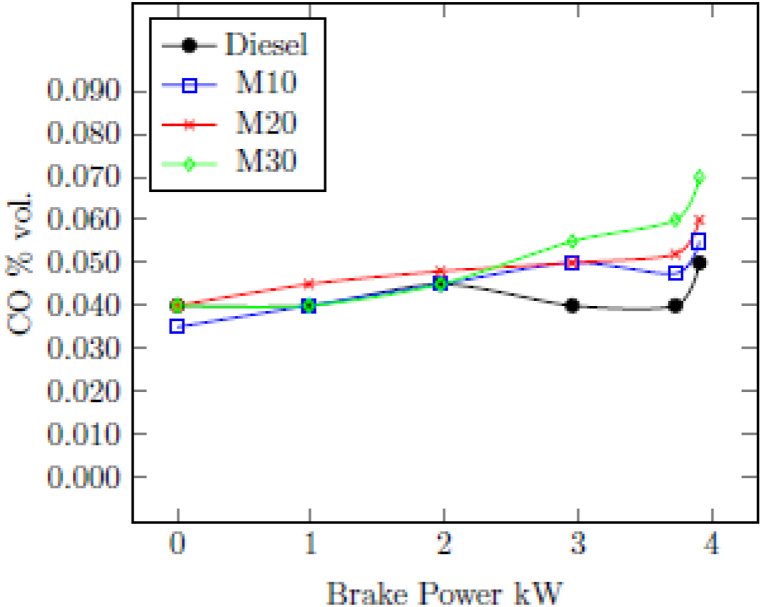
Change in CO emission with BP.

Fig. 3 illustrates the variation in CO in a diesel engine running on M10, M20, M30, and pure diesel under various load conditions. For all fuels, there is no discernible variation in CO emissions at low engine loads. Diesel was found to have CO emissions of 0.05%vol compared to 0.055%vol with M10, 0.052%vol with M20, and 0.06%vol with M30 at the maximum load of 3.73 kW. Even though there is a slight increase in CO emission for blended fuels because of deterioration of combustion due to high latent heat vaporization of ethanol present in the emulsion which is responsible for the poor oxidation reaction rate of CO. In addition, the presence of oxygen in the ethanol participates oxidation of CO causes comparable CO emissions of blended fuels with diesel. The higher CO emission with M30 in peak engine load is due to the episode of reduced temperature combustion which follow the formation of OH radicals with the dissociation of water and further react with carbon to form CO [42].

#### 3.2.2 Carbon dioxide (CO_2_)

**Fig. 4:**
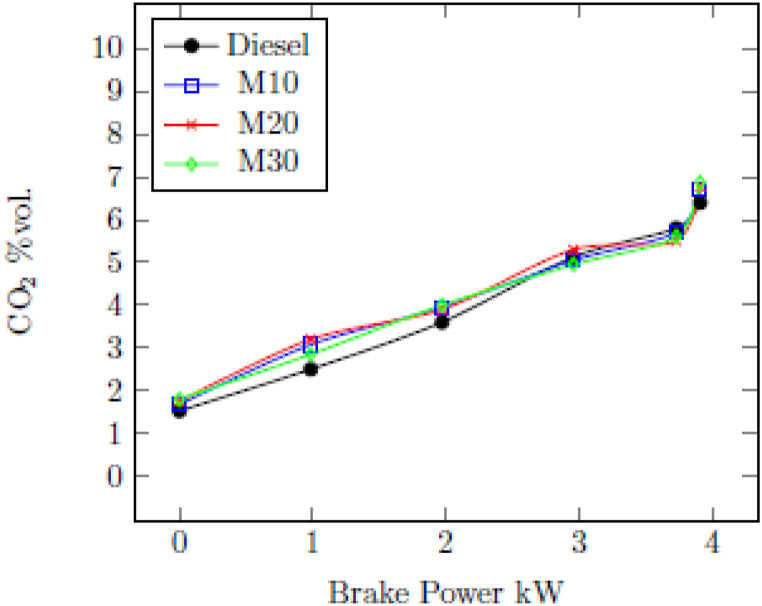
Change in CO_2_ emission with BP.

Fig. 4 indicates the change in CO_2_ emissions of tested engine with M10, M20, M30 and diesel for different loads. For various fuels, the CO_2_ increased as the load increased. Diesel was found to have a CO_2_ of 5.8%vol. at the highest load of 3.73 kW, compared to 5.7%vol. with M10, 5.5%vol. with M20, and 5.6%vol. with M30. Even though a small decrease in CO_2_ emissions with blended fuels could be stated as the deterioration of the combustion due to the presence of ethanol, which has high latent heat of vaporization lead to poor oxidation of CO to CO_2_. In addition, the presence of oxygen in ethanol in M10, M20 and M30 leading the oxidation process of CO to CO_2_ make comparable CO_2_ emissions with diesel.

#### 3.2.3 Nitric oxide (NO)

**Fig. 5:**
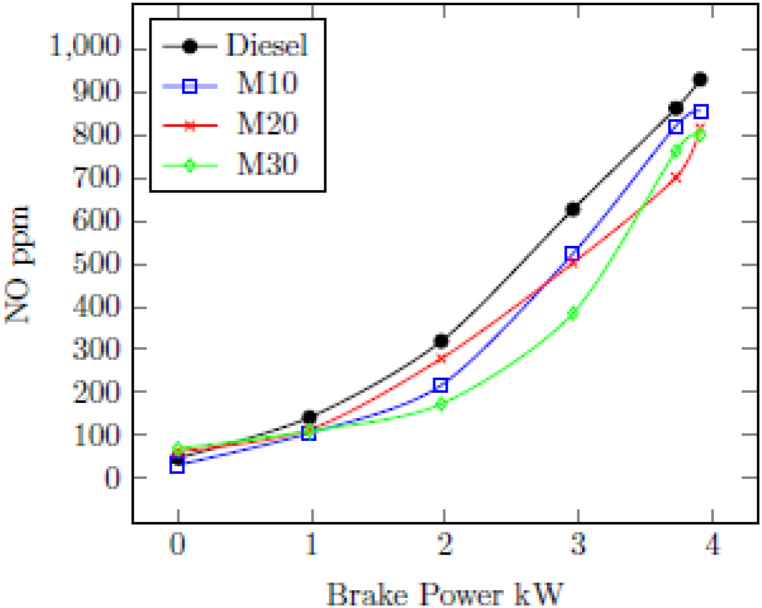
Change in NO with BP.

Fig. 5 indicates the change in NO fuelled with M10, M20, M30 and diesel for different loads. NO are start to increase along load for all fuels due to higher in-cylinder temperature. Diesel had a NO of 862 ppm with highest load of 3.73 kW, compared to 822 ppm with M10, 707 ppm with M20, and 763 ppm with M30. The NO was lower with all blended fuels owing to the high latent heat of vaporization of ethanol in the emulsions further reduces the in-cylinder temperature. In addition, the evaporation of water component in the microemulsion blends absorbs large amount of heat further reduces the peak flame temperature during the combustion leading reduced emissions of NO. Finally, the presence of water and alcohol in the blended fuels causes reduced NO emission [26].

#### 3.2.4 Exhaust gas temperature (EGT)

**Fig. 6:**
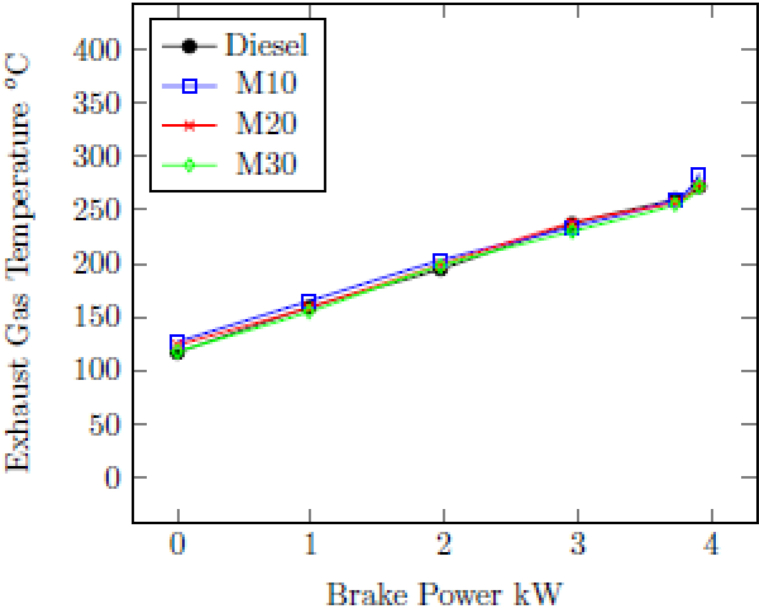
Change in EGT with BP.

Fig. 6 indicates the change in EGT with BP for M10, M20, M30 and diesel for different loads. At higher engine loads increased EGT was observed due to the increased fuel burning of fuel. EGT of 270^o^C was recorded with diesel at the highest load of 3.73 kW, compared to 265^o^C with M10, 257^o^C with M20, and 255^o^C with M30. The lesser EGT for emulsified fuel blends are owing to the high latent heat of vaporization of water and ethanol present in M10, M20 and M30 lowers the burning temperature. The lesser EGT with fuel blends are due to their lower calorific value in comparison with diesel [43].

#### 3.2.5 Unburnt hydrocarbon (UBHC)

**Fig. 7:**
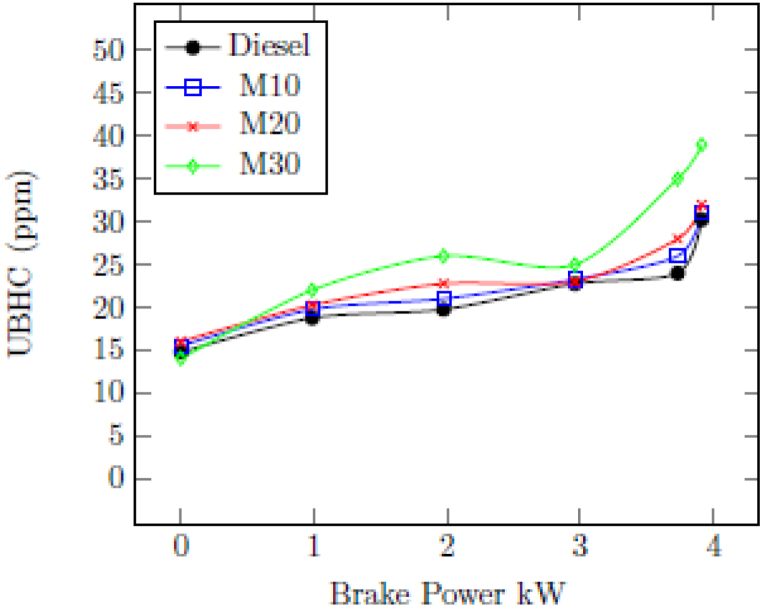
Change in UBHC with BP.

Fig. 7 indicates the change in UBHC of tested engine with M10, M20, M30 and diesel for different loads. UBHC of 24 ppm was recorded with diesel as that of 26 ppm, 28 ppm, and 35 ppm, respectively, for M10, M20, and M30 at the maximum load of 3.73 kW. The presence of an unburned ethanol quench layer in the combustion chamber could be attributed to the increase in UBHC associated with emulsified fuels at lesser engine loads. Furthermore, the high latent heat of water vaporization produces slower vaporization and increased UBHC emissions may have resulted from fuel and air mixing. Additionally, the lack of homogeneity in M10, M20, and M30 combined with their higher viscosity and lower volatility results in higher UBHC emission. The increase in UBHC emission with blended fuel is caused due to higher ignition delay as shown in Fig. 11 [44].

**Fig. 8:**
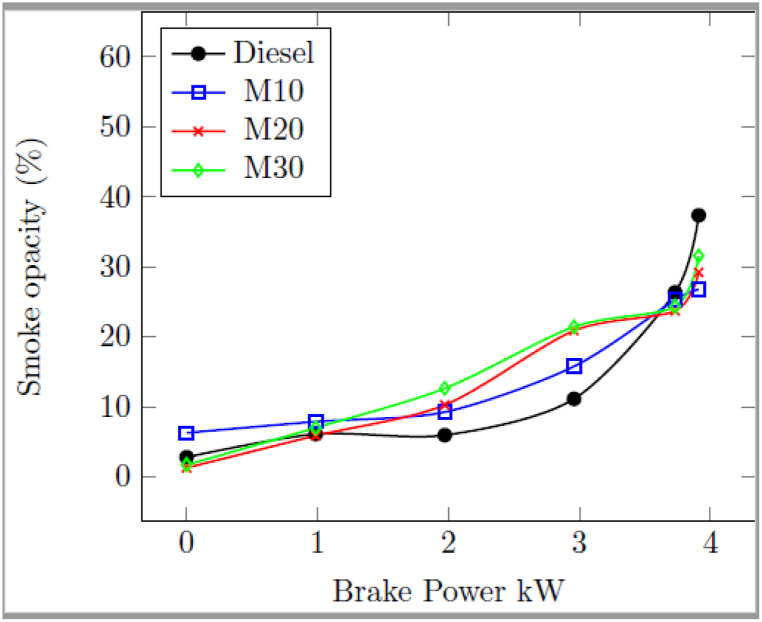
Change in smoke opacity with BP.

**Fig. 9:**
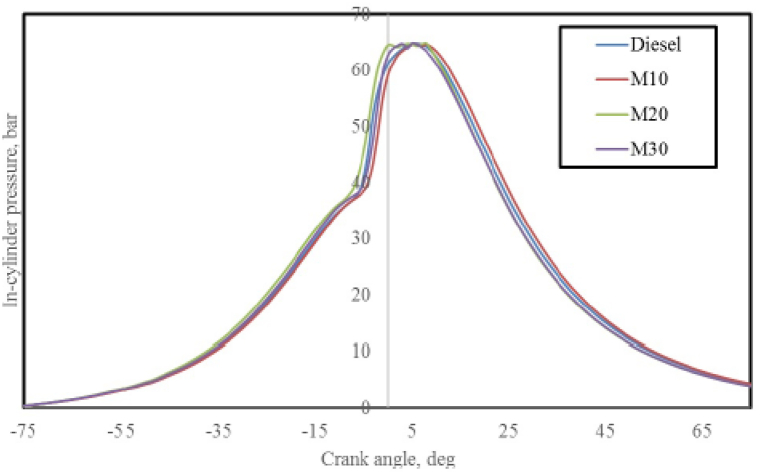
Change in in-cylinder pressure with BP.

**Fig. 10:**
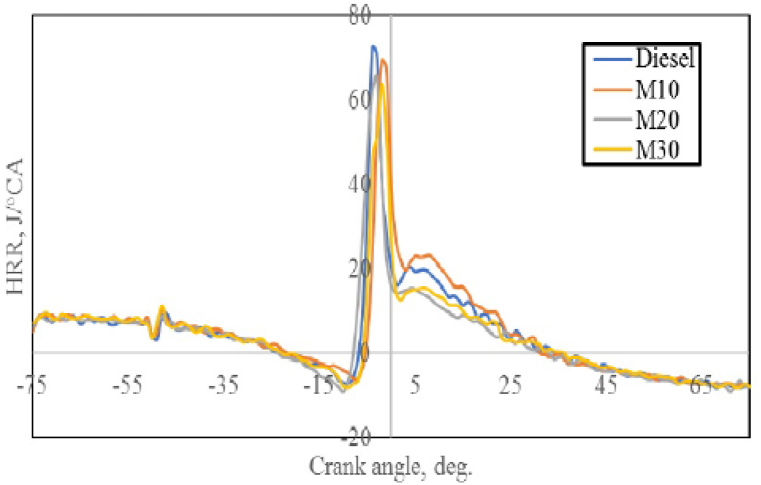
Change in HRR with BP.

**Fig. 11:**
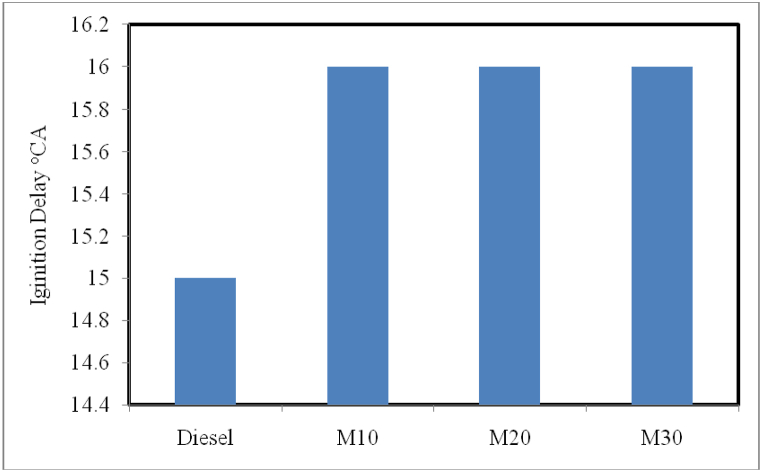
Change in ignition delay with fuel type.

#### 3.2.6 Smoke opacity

Fig. 8 indicates the change in smoke opacity of tested engine with M10, M20, M30 and diesel as fuels for different loads. At highest load of 3.73 kW, 26.2% smoke opacity with diesel as compared to 25% with M10, 23.5% with M20 and 24.4% with M30, which was due to the presence of ethanol in the emulsion, oxidizes the fuel rich regions and suppresses the soot formation in the combustion chamber. In addition, the high oxygen content of emulsion with low C/H ratio and low aromatic fractions contributes reduction of smoke with blended fuel as compared to diesel. In addition, a significant amount of air entrainment in the emulsion spray, a micro-explosion phenomenon, and an increase in spray volume were the causes of the decreased smoke opacity for blended fuel at 3.73 kW [43].

### 3.3 Characteristics of combustion

#### 3.3.1 In-cylinder pressure

The average in-cylinder pressure variation for the M10, M20, and M30 as well as pure diesel at 3.73 kW is shown in Fig. 9. Diesel was found to have the peak in-cylinder pressure of 64.91 bar, compared to 64.1 bar for M10, 63.79 bar for M20, and 63.16 bar for M30. Peak pressures with emulsified blends and diesel differed from one another. It was discovered from the literature that diesel has a higher cetane number than microemulsion. [31], hence ignition delay is prolonged with the superior premixed burning rate which could reduce the peak pressures of blends as compared to diesel. In addition, the higher density of blended fuels causes advancing of dynamic injection timing. The fuels ability to through mixing with air greatly affected by in-cylinder pressure [42]. A marginal deviation in the in-cylinder pressures of blended fuels and diesel indicates its competence as fuel.

#### 3.3.2 Heat release rate (HRR)

Fig. 10 indicates the change in HRR with crank angle for M10, M20, M30 and diesel when the engine is working in highest load. The HRR negative is observed for all fuels before combustion could be due to chilling effect of cylinder charge caused by the vaporization of accumulated fuel in the ignition delay period which also reported by [42], whereas the HRR becomes positive subsequent to the beginning of combustion. The negative HRR was due to the chilling effect caused due to the absorption of heat from the combustion chamber with the vaporization of fuel injected inside the combustion chamber. The increased density of emulsified fuel blends makes it possible for fuel to be injected ahead of schedule thanks to advances in dynamic injection timing. This results in a longer ignition delay because more fuel builds up inside the combustion chamber. Diesel was found to have a higher HRR of 72.30 J/^o^CA as opposed to 69.41 J/^o^CA with M10, 66.22 J/oCA with M20, and 64.77 J/^o^CA with M30. Table 2 illustrates that the microemulsion blends' increased kinematic viscosity and decreased calorific value caused the decrease in HRR. For fuels blended with microemulsions, the higher kinematic viscosity results in less fuel/air mixing during injection, which lowers HRR.

#### 3.3.3 Ignition delay

Due to its poorer ignition quality, *S. molesta* oil microemulsion blends were shown to have an increased ignition delay (ID) when compared to diesel. As indicated in Fig. 11, the lower cetane number of microemulsion blends leads to higher ignition delay as reported by [31]. This causes major impact on its lower ignition quality and increases the premixed burning rate, which leads to comparable peak in-cylinder pressure with diesel. In addition, the increase in ID of the emulsified blends was due to vaporization of ethanol and water causing relatively low gas temperature environment to the injected fuel spray. This causes longer time for the vaporization and mixing of emulsified blends further increases the ignition delay.

## 4 Conclusions

The microemulsion produced from *S. molesta* was blended with diesel as M10, M20 and M30 and its properties are characterized and compared with diesel. Experiments were performed to analyze the performance, emission and combustion analysis of diesel engine fuelled with blends of *S. molesta* micro emulsion with diesel. The conclusions of this study as follows:

• *S. molesta* microemulsion's physical and chemical characteristics were comparable to those of diesel and the ASTM 6751-02 standard, which investigates its potential use as a substitute fuel for stationary diesel engines.
• The performance evaluation of a diesel engine running on M10, M20, M30, and diesel produced a minimum BSFC of 0.2745 kg/kWh for diesel and a maximum BTE of 30.71%. the modest BTE decrease seen with M10, M20, and M30. The BTE for M10 is reported to be 30.06%, while at a highest load of 3.73 kW, M20 has a slightly lower BTE of 29.76%.
• At a highest load of 3.73 kW, the exhaust emissions produced a marginal increase in CO, CO_2_, and unburned hydrocarbon of 3.8%, 3.4%, and 16.66%, respectively, with M20 being that of diesel. NO and smoke were reduced by 18.07% and 7.37%, respectively.
• M20 has a lower peak in-cylinder pressure and heat release rate than diesel, by 1.73% and 8.40%, respectively, based on the analysis of combustion parameters.
• Ultimately, these findings investigate the potential of S. molesta microemulsion as a substitute in CI engines; of all the blends, M20 was found to be the best, with a minor decrease in BTE when compared to M10 and diesel, as well as a decrease in NO and smoke emissions. As future work adding suitable nano particles with the microemulsion along with a variable compression ratio multi-fuel diesel engines further investigations can be conducted by considering different parameters.

## Data availability statement

The authors certify that the data pertaining to this manuscript is made available on request.

## CRediT authorship contribution statement

**Marutholi Mubarak:** Writing – original draft, Methodology, Investigation, Conceptualization. **Andavan Shaija:** Writing – review & editing, Supervision, Project administration, Funding acquisition. **Tharamel Vasu Suchithra:** Writing – review & editing, Supervision, Funding acquisition.

## Declaration of competing interest

The authors declare that they have no known competing financial interests or personal relationships that could have appeared to influence the work reported in this paper.
